# Health System Resilience as the Basis for Explanation Versus Evaluation: Comment on "The COVID-19 System Shock Framework: Capturing Health System Innovation During the COVID-19 Pandemic"

**DOI:** 10.34172/ijhpm.2023.7481

**Published:** 2023-02-26

**Authors:** Stephanie M. Topp

**Affiliations:** College of Public Health Medical and Veterinary Sciences, James Cook University, Townsville, QLD, Australia.

**Keywords:** Health Systems, Resilience, Power, Explanatory Research, Evaluative Research, Systems-Thinking

## Abstract

The onset and impacts of COVID-19 have prompted attention to national health system preparedness for, and capacity to adapt in response to, public health emergencies and other shocks. This preparedness and adaptive capacity are often framed as ‘health system resilience’ a concept previously associated more with assessments of health systems in conflict-affected and fragile states. Yet health system resilience remains a slippery concept, defined and applied in multiple ways. Reflecting on the Hodgins and colleagues’ study "*the COVID-19 system shock framework: capturing health system innovation during the COVID-19 pandemic*," this article restates the limitations of health systems resilience as a concept capable of anchoring evaluative assessments of health system performance but stresses its value in the context of explanatory research investigating *how* and *why* health systems adapt, with due attention to the power of actors’ whose choices inform the nature and direction of change.

 The onset and now deep impacts of COVID-19 and associated lockdowns have prompted attention to national health system preparedness for, and capacity to adapt in response to, public health emergencies and other shocks.^1-3^ This preparedness and adaptive capacity are often framed as ‘health system resilience’ a concept previously associated more with assessments of health systems in conflict-affected and fragile states^4^; but more recently incorporated into analyses of a much broader range of health system settings including in middle and high income countries.^5,6^ Hodgins and colleagues’ study “*The COVID-19 system shock framework: capturing health system innovation during the COVID-19 pandemic,”* set in a large paediatric health service network in Sydney, Australia, represents an example of this uptake with the authors presenting an adapted framework to explore how and why their service network responded to COVID-19 related shocks in certain ways.^7^

 A valuable feature of the concept of resilience in the context of health research is its promotion of systems-thinking^8^; a holistic approach to analysing how components of health systems interact and adapt.^9^ Although health policy and systems researchers with multi-disciplinary training have for some time advocated the need for systems-thinking as core to improving health system performance, engagement with and uptake of the tools and methodologies in health services research more generally has been slow.^10^ In part this is because neither the tools nor methodologies are part of the traditional health and medical research training curricula. And in part it is the result of an enduring focus among medical practitioners and researchers on the material inputs and technical functions that are the most visible aspects of health systems, discounting the critical nature of the social, relational and political factors that drive health system performance.

 In that context, the concept of health system resilience has served as a useful entry point for a broader range of researchers and policy makers to explore *how* and *why* change occurs within a health system. Since health system resilience is popularly defined as a health system’s capability to absorb, adapt or transform in order to prepare for and respond to a shock or shocks,^11^ this emphasis on the explanatory power of the concept is key.

 In Hodgins and colleagues’ study the authors adapt Hanefeld et al^12^ ‘learning from shocks’ framework drawing on the World Health Organization (WHO) Building Blocks to adapt the functional components to include health services, health workforce, information systems, products and technologies and funding and finance.^7^ Health system values, and health policy and governance are presented as cross cutting domains informing the direction and nature of responses within the five functions. The framework provides a heuristic for organizing and reflecting on changes to the modes and outputs of a service network in response to an (external) shock, and asks questions regarding how and why certain activities and innovations occur within each of the five functional domains. Whether this constitutes a robust evaluation of health system (or in this case *service-network*) resilience is less clear, however, in part because it remains unclear whether the authors understand health system resilience as an outcome or an ability, and relatedly, whether the framework is designed to support explanatory or evaluative research.^13^

 In the framework presented, *health system resilience* is placed in a box to the right of the five functional domains in a manner that implies it is the outcome of a value driven, well-governed system. The implication is that resilience – a normative good – is the product of adaptive responses, guided by values and governing institutions of the system. Indeed a significant portion of the results is given over to describing innovations – granular changes to service planning or modes of service activity within the network – across the functional and cross-cutting domains, demonstrating various examples of absorptive and adaptive change. The implication of both the framework and the results is that adaptation itself is illustrative of (the presumably positive outcome) health system resilience. Yet as the authors acknowledge (p. 10), evaluation of those innovations’ impact on service or health outcomes, in particular for the children and carers who make up the network’s primary service users, was not part of the study.

 This challenge – descriptions of adaptions made in the face of some shock, but absent an evaluation of their impact on quality, coverage or cost – is revealing of several key dangers associated with using health system resilience in evaluative work. That is, in the concept’s inability to account for either the direction (positive or negative) of expected change; or the basis on which such change occurs; as well as whether such adaptations have produced *un*expected change in the context of what are famously non-linear health systems. In Hodgins et al, the authors provide a rich account of the network’s response to COVID-19 shocks including key examples of absorptive and adaptive change. The authors demonstrate the capability of network *agility* grounded in disseminated leadership and effective management that enabled rapid adaptations in service and workforce functions. The capability of *intelligence *was also implied in the descriptions of the well-resourced information systems and leadership decisions to enact bi-directional information sharing that helped manage (forecasted) fear and confusion among frontline providers, and equally, helped to ensure leadership were learning from the experiences of frontline experiences. Against a backdrop of limited COVID-19 case numbers but significant operational pressures linked to state-wide public health directives, the robust resourcing of the network and access to additional funds were also central to its absorptive capacity – including by enabling rapid mobilization of special teams; ensuring IT capabilities to support conversion to telehealth services; and underpinning workforce capacity to generate standard operating procedures. These are intuitively positive experiences and indicative of adaptive capabilities, but quite distinct from evidence of system-wide *benefit* which is the implication of the framework, if not the qualified findings presented. Looking at the direction of expected change, and exploring unexpected changes across different timelines and domains of a health system through other means of evaluation is thus crucial.

 Elsewhere in the rapidly expanding health systems resilience literature the reverse situation may be observed, in which health system performance indicators (eg, service coverage, or medicines availability) are used as a proxy for evaluating the ‘outcome’ of health system resilience.^14^ Good performance (based on cross-sectional or trending indicators) is sometimes equated with a system capable of being resilient or bouncing back from some shock. But without the complementary work to explain how and why that performance is achieved, this type of evaluation too, is flawed. It misses the possibility (even likelihood) that good performance can occur off the back of a brittle system lacking robustness^15^ and overly reliant on certain individuals or groups (cf Lee et al^16^); or conversely, the possibility that *failure* to reach target indicators or improve system performance is linked to deliberate choices made by actors who have an interest in maintaining the status quo.^17,18^ The problematic nature of framing health system resilience as an outcome in this way lies at the heart of a long-standing critique of the concept as being power blind.^13,19^

 One way of gauging whether initiatives or innovations are power-sensitive or power-blind is to consider the degree to which they target the causes, versus the symptoms, of a system-wide challenge. Where innovations require more effort from those with least power in a system, shifting the burden of adjustment onto individuals or groups with no option but to simply ‘cope,’ innovations are likely to be power-blind. In this respect, Hodgins et al and the COVID-19 System Shock Framework demonstrate some power sensitivity, specifically calling attention to the importance of identifying which health system values inform and direct decisions of those in power and describe instances of both both power-sensitive and power-insensitive innovations. The use of simulations to disseminate critical information embedded in newly developed standard operating procedures, demonstrated sensitivity to the inherent time constraints of frontline staff and the need to provide opportunities for active (team based) learning in order operationalise new modes of care delivery. The democratisation of access to real-time data on COVID-19 cases, their management and key commodities via the dashboard is another example of an innovation drawing on centralised resources to improve and ease the work of stretched frontline staff. Conversely, descriptions of the innovations around telehealth included reports of a significant additional burden on providers and (at least in the context of this study) less well documented evidence of benefit for service users. Such circumstances are suggestive of a power-insensitive innovation requiring careful assessment of the degree to which frontline staff are required to absorb the additional cognitive and emotional workload of telehealth as well as the true benefits in terms of service and health outcomes.

 Hodgin and colleagues’ work reminds us of the conceptual slipperiness of health system resilience, and both the strengths and pitfalls of its analytical value to health policy and systems researchers. On the one hand, the article demonstrates the usefulness of resilience as an entry point for systems-thinking, incorporating integrated analysis of the way critical material (service, health workforce, medical products) and relational (values, leadership, communication) components of a health system operate as a dynamic whole. On the other hand, the authors themselves acknowledge the challenges of using health system resilience in a quasi-evaluative manner absent the integration of other types of measurement. A key take away from this article is the productive role of health system resilience as a concept to enhance *explanatory* accounts of change (or resistance to it), but concurrent limitations with regard *evaluative* assessments of health system performance.

## Ethical issues

 Not applicable.

## Competing interests

 Author declares that she has no competing interests.

## Author’s contribution

 SMT is the single author of the paper.

## Funding

 SMT holds a NHMRC Investigator Award (2020-24) GNT1173004.
